# Persistence of *Babesia microti* Infection in Humans

**DOI:** 10.3390/pathogens8030102

**Published:** 2019-07-17

**Authors:** Evan M. Bloch, Sanjai Kumar, Peter J. Krause

**Affiliations:** 1 Johns Hopkins University School of Medicine; Baltimore, MD 21287, USA; 2 Food and Drug Administration, Silver Spring, MD 20993, USA; 3 Yale School of Public Health and Yale School of Medicine, New Haven, CT 06520 USA

**Keywords:** *Babesia*, *Babesia microti*, persistence, recurrence, *Plasmodia*, malaria, spleen, blood transfusion

## Abstract

Persistent infection is a characteristic feature of babesiosis, a worldwide, emerging tick-borne disease caused by members of the genus *Babesia.* Persistence of *Babesia* infection in reservoir hosts increases the probability of survival and transmission of these pathogens. Laboratory tools to detect *Babesia* in red blood cells include microscopic detection using peripheral blood smears, nucleic acid detection (polymerase chain reaction and transcription mediated amplification), antigen detection, and antibody detection. *Babesia microti*, the major cause of human babesiosis, can asymptomatically infect immunocompetent individuals for up to two years. Chronically infected blood donors may transmit the pathogen to another person through blood transfusion. Transfusion-transmitted babesiosis causes severe complications and death in about a fifth of cases. Immunocompromised patients, including those with asplenia, HIV/AIDS, malignancy, or on immunosuppressive drugs, often experience severe disease that may relapse up to two years later despite anti-*Babesia* therapy. Persistent *Babesia* infection is promoted by *Babesia* immune evasive strategies and impaired host immune mechanisms. The health burden of persistent and recrudescent babesiosis can be minimized by development of novel therapeutic measures, such as new anti-parasitic drugs or drug combinations, improved anti-parasitic drug duration strategies, or immunoglobulin preparations; and novel preventive approaches, including early detection methods, tick-avoidance, and blood donor screening.

## 1. Introduction

*Babesia* are intraerythrocytic protozoa in the same Apicomplexa phylum as *Plasmodia*. They are transmitted worldwide by hard-bodied ticks and infect a wide variety of wild and domestic animals. Several *Babesia* species have been found to infect humans, the most important of which is *Babesia microti*, which is endemic in the Northeastern and northern Midwestern United States [1]. The number of cases and geographic range of *B. microti* have increased over the past two decades in endemic areas. About 2000 cases of *B. microti* are reported each year but epidemiologic studies suggest that the actual number of cases is much greater [1,2,3]. *B. microti* also can be transmitted through blood transfusion, organ transplantation, and perinatally [1,4]. It causes a malaria-like illness that is fatal in about 20% of immunocompromised hosts and those who acquire the infection through blood transfusion.

Persistent infection is a characteristic feature of babesiosis and malaria, the two primary intraerythrocytic protozoal diseases of humans [5,6,7,8,9]. Both depend on an arthropod vector to transmit infection from other humans (*Plasmodia*) or animal hosts (*Babesia*). Persistence of infection in the reservoir host increases the probability of transmission and survival of these pathogens [6]. Chronic malaria infection has long been recognized. Recrudescence of *Plasmodium vivax* and *Plasmodium ovale* infections have been reported more than two and four years after initial infection, respectively [8]. Human babesiosis also has been documented to persist and recrudesce. Among immunocompetent hosts, *Babesia microti* parasitemia (DNA) has been detected for more than a year in patients treated with a standard course of antibiotic therapy and more than two years in untreated individuals [10,11]. Immunocompromised patients can experience persistent *B. microti* parasitemia and relapsing symptoms for more than two years despite antimicrobial therapy [12,13]. Both *Plasmodia* and *Babesia* reside within red blood cells, which are somewhat impervious to immune attack. Furthermore, *P. ovale* and *P. vivax* also sequester within liver cells during the hypnozoite stage of the life cycle. In this paper, we will focus on persistence of *B. microti* infection, the most common cause of babesiosis in humans. We will examine laboratory tools that have been used to document persistence of *Babesia* infection (including blood smear, PCR, animal inoculation, and antibody), asymptomatic and symptomatic persistence of infection following tick-transmitted infection, asymptomatic persistence of infection in blood donors, and mechanisms of *Babesia* persistence.

## 2. *Babesia microti* Detection Tools

**Figure 1:**
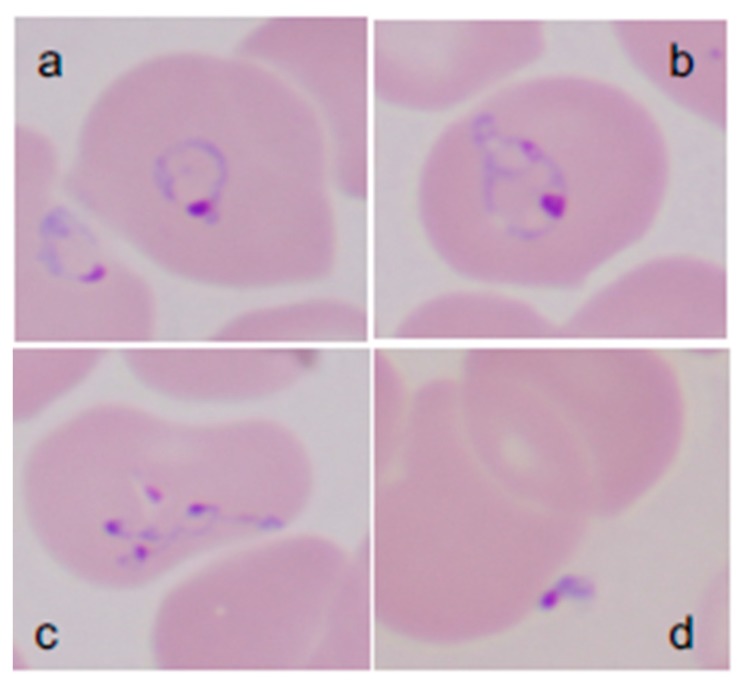
**Giemsa-stained thin blood films showing *Babesia microti* parasites.***B. microti* are obligate parasites of erythrocytes. Trophozoites may appear as ring forms (**A**) or as ameboid forms (**B**). Merozoites can be arranged in tetrads and are pathognomonic (**C**). Extracellular parasites can be noted, particularly when the parasitemia level is high (**D**). (Adapted from [1]).

The diagnosis of babesiosis should be considered in anyone who resides in or travels to a *Babesia* endemic area or has received a blood transfusion within the previous six months and has typical clinical symptoms, such as fever, fatigue, malaise, weakness, chills, sweats, and headache. The diagnosis is confirmed through identification of *Babesia* on microscopic examination of Wright or Giemsa-stained thin blood-film or detection of *Babesia* nucleic acid using such methods as polymerase chain reaction (PCR) (Figure 1) [1,14,15]. *Babesia* and *Plasmodium falciparum* are morphologically similar on microscopic examination. Features that help to discriminate between the two include the lack of hemozoin pigment deposits and the presence of tetrad or “Maltese cross” forms in *Babesia*-infected erythrocytes. The latter are rarely seen but when identified are pathognomonic for *Babesia* infection. The sensitivity of blood-film microscopy for *Babesia* detection is about 85%, while specificity approaches 100% [15,16]. Review of multiple thick and thin-blood films increases sensitivity of microscopic detection. Microscopy is suboptimal for the detection of low-level parasitemia as occurs both in early and long-term chronic infection and is therefore not considered an effective tool for epidemiological and surveillance purposes.

Nucleic acid detection offers a better correlate of active infection. Nucleic acid detection based tests (NAT), such as polymerase chain reaction (PCR) and transcription-mediated amplification (TMA), more effectively identify low-level infections than other laboratory tests [15,16,17,18,19]. The molecular methods for *Babesia* detection generally rely on amplification of the 18S gene, which encodes the small subunit ribosomal RNA gene. Publication of the full *B. microti* genome in 2012 has enabled identification of higher copy number detection targets, allowing the routine detection of less than 10 *B. microti* parasites per ml of blood [18,20]. Additional technological advances such as sample concentration, high copy detection targets such as BMN multigene family members, and bead-based target capture may further enhance the sensitivity of NAT assays [21,22]. Detection of *Babesia* antigen(s) offers additional markers of active infection. Efforts should be made to identify excreted/secreted *B. microti* antigen to develop high throughput detection assays for diagnostic and donor screening purposes [18,22]. Inoculation of patient blood into small mammals such as hamsters, gerbils, or SCID mice for detection of *Babesia* is less sensitive than PCR and impractical but is useful for investigation of rare variants/strains of *Babesia* and for blood donor studies [15].

Antibodies are the most reliable marker of past exposure to the *Babesia* parasite. The indirect fluorescent antibody (IFA) assay is the most commonly used *Babesia* antibody test, which utilizes the whole parasite as an antigen source [15,23]. The CDC defines a *Babesia* laboratory supportive antibody test result as an IFA total immunoglobulin or IgG antibody titer of ≥1:256 (or ≥1:64 in epidemiologically linked blood donors or recipients) [24]. An enzyme-linked immunosorbent assay (ELISA) test also has been developed for detection of antibodies to the immunodominant *B. microti* antigens BMN-9 and BMN-17 [25]. A commercial ELISA based on the synthetic peptides representing these antigens was used for screening blood donors for anti-*B. microti* antibodies in investigational studies [26,27]. Recent advances, which include genomics-based approaches for antigen discovery, bead particle-based antigen multiplexing, and application of nanotechnology, are all anticipated to improve the sensitivity of *Babesia* antibody-based assays [28,29]. A major limitation of antibody-based assays is that a single antibody result fails to distinguish between present (active) and past (resolved) infection, although a *B. microti* IgG antibody titer of ≥1:1024 or the presence of IgM antibody suggest recent infection [30,31]. A four-fold rise in *Babesia* antibody confirms recent infection but is impractical because convalescent sera must be obtained at least two to three weeks after sera is first obtained during acute illness (acute sera). Identification of antigens that could distinguish between active and resolved infections would greatly enhance the utility of antibody-based assays for *Babesia* diagnosis.

## 3. Persistent *Babesia microti* Infection in Immunocompetent and Immunocompromised Hosts

**Figure 2:**
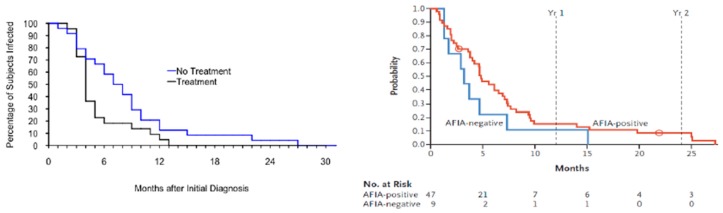
**Persistence of *B. microti* infection.***Left panel*. Persistence of *Babesia microti* DNA in humans following acute babesiosis. Blood samples from patients experiencing acute babesiosis were tested for *B. microti* DNA using PCR every three months following the onset of infection until DNA was no longer detectable. Panel shows the Kaplan–Meier estimate of the survival function modeling the time to the first PCR-negative follow-up sample among study subjects with *B. microti* infection (adapted from [10]). *Right panel.* This panel shows the Kaplan–Meier estimate of the survival function modeling the time to the first PCR-negative follow-up sample in blood donors whose samples were PCR positive for *B. microti* on the index blood donation sample. The results of two groups are shown, those that were *Babesia* antibody positive as determined by AFIA (semi-automated arrayed fluorescent immunoassay) and those who were *Babesia* antibody negative (adapted from [11]).

Persistent *Babesia* infection was first reported in an immunocompetent 59-year-old woman who contracted the infection on Nantucket, Massachusetts [32]. She experienced *Babesia* symptoms for three weeks and *B. microti* parasites continued to be detected on blood smear for more than four months after symptoms resolved. The first prospective study of persistent babesiosis was carried out in 46 residents of southern New England [10]. Blood samples were obtained from patients during acute infection and every three months thereafter for amplification of *B. microti* DNA using PCR. Asymptomatic parasitemia was shown to last more than a year in a few patients, despite treatment with clindamycin and quinine. Asymptomatic infection persisted even longer in a group of patients who had mild or subclinical *B. microti* infection and were not treated because of concern about side effects of clindamycin and quinine. One untreated patient had persistence of infection and recrudescence of symptoms 27 months after diagnosis [6]. Similar results were noted in another study of 56 asymptomatically infected blood donors (Figure 2) [11].

Prolonged *Babesia* disease has been described in immunocompromised hosts, with relapsing symptoms lasting up to nine months and parasitemia continuing for more than a year [33,34,35,36]. These patients were markedly immunocompromised given underlying diagnoses that included HIV/AIDS, malignancy, and asplenia. A retrospective case series of consecutively enrolled babesiosis patients who failed to respond to standard anti-*Babesia* antibiotic therapy also demonstrated that patients with these immunosuppressive conditions experienced persistent and relapsing babesiosis [12]. Interestingly, 10 of the 14 patients in this series suffered from B cell lymphoma and had been treated with Rituximab, an anti-B cell monoclonal antibody. These data suggest that an impaired anti-*Babesia* antibody response, in the context of generalized immunosuppression, prevents clearance of *B. microti* infection. Long term antibiotic therapy of at least six weeks, rather than the standard 7 to 10 days, was required to resolve infection in these severely immunocompromised patients [12]. Severe and persistent *B. microti* infection also has been associated with advanced age in a mouse model and in humans [1,36,37].

## 4. Persistent *Babesia microti* infection in Asymptomatic Immune-Intact Blood Donors

*Babesia* can be transmitted through blood transfusion and has long been recognized as a leading infectious risk to the blood supply in the United States, particularly in parts of the Northeast and Upper Midwestern states where *B. microti* is endemic [38]. To date over 200 cases of transfusion-transmitted babesiosis (TTB) have been described [39], the overwhelming majority of which have been caused by *B. microti* [4]. In addition, rare cases of TTB have been attributed to other species (e.g., *Babesia duncani*) [40] and variant *Babesia* strains [41]. While *Babesia* species are globally ubiquitous, findings from the few studies outside of the US that have investigated blood donors have failed to match the scale encountered here [1,42,43,44]. 

*Babesia* is transmissible through any red blood cell containing blood product, including packed red blood cells (RBCs), whole blood, and whole blood-derived platelets [4,45]. Findings from a murine model suggest that as few as 10 to 100 RBCs are needed to establish host infection [46]. Transfusion recipients are at high risk for severe or complicated disease. For one, there is overrepresentation of vulnerable patient subsets among transfusion recipients such as those at extremes of age, those with asplenia, or those who have just undergone surgery. Furthermore, the primary indication for red blood cell transfusions is anemia, which exacerbates underlying disease and compounds the risk of complications. Such is reflected by the high fatality rate of TTB (19%) [4]. 

Unlike natural (i.e., tick-borne) acquisition, TTB is not strictly seasonal. Prolonged storage of blood components enables transfusion of parasitemic blood long after blood donation. In addition, the incubation period for development of symptoms after transfusion is as long as six months. TTB is also not geographically bound, given that blood donors from non-endemic areas may travel to endemic areas where they can acquire the infection through tick bite, return to the non-endemic area and donate infectious blood. Finally, blood that is collected in endemic states is frequently shipped to states that are not considered high risk for babesiosis, also accounting for cases of TTB in non-endemic states [47,48,49]. 

The risk of TTB is greatly increased by persistent asymptomatic parasitemia in blood donors [11,50,51,52]. Blood donors are an invaluable resource to study the kinetics of *Babesia* infection in healthy individuals. The donation process itself selects for asymptomatic individuals. Those with high parasitemia would be expected to manifest symptoms, and either self-defer from blood donation or be deemed ineligible given detectable fever, low hemoglobin and/or abnormal vital signs that would likely be detected during pre-donation screening. Historically, individuals who reported a history of babesiosis were permanently deferred from blood donation, further emphasizing that donors are typically unaware of prior and/or ongoing *Babesia* infection at the time of donation. 

Recognition of risk to the US blood supply spurred a series of studies to characterize the burden and immunopathogenesis of *Babesia* in blood donors. These studies revealed a high *B. microti* seroprevalence in endemic areas (up to 2.5% in parts of Connecticut) with molecular (PCR) evidence of infection in up to a half of seropositive donors [3,19]. 

About a fifth (21%) of 84 seropositive blood donors (IFA titers ≥64), who were followed for up to three years in Connecticut and Massachusetts were found to be parasitemic [50]. Over the course of follow-up, protracted low-level parasitemia was variably and intermittently detectable. One subject was found to be parasitemic on initial screening and then again 13 months later, as shown by hamster inoculation. Those who were parasitemic were similar with respect to demographic and geographic characteristics to those who were not, but displayed higher *Babesia* IFA antibody titers in aggregate (median IFA titer ≥256 vs. ≥64). 

Development of blood donor screening assays, which began in the mid-to-late 2000s, largely through partnerships between small businesses and the major blood collection agencies in the US, expanded testing capacity and further enabled the study of *Babesia* kinetics in the donor population. In one study over a two-year period (June 2012–September 2014), a total of 89,153 blood-donation samples were tested with a semi-automated arrayed fluorescent immunoassay (AFIA), together with a PCR assay. A total of 335 (0.38%) samples were shown to be seroreactive by AFIA, 67 (20%) of which were also PCR positive [52] (Figure 1). Furthermore, 1 per 9906 screened samples was seronegative but PCR positive. Approximately a third of the red blood cell samples from PCR positive or high-titer AFIA seroreactive donations resulted in infection following inoculation into hamsters. After a year of follow-up, DNA persistence based on PCR was noted in 14% of the donors. In contrast, 92% of donors displayed evidence of antibody persistence. 

During validation studies of a new *B. microti* enzyme-linked immunoassay (ELISA) in 15,000 blood donors, the investigators reported 1.08% seroreactivity in those residing in a highly endemic area (Long Island, NY) [22]. In a subsequent study in which blood donors were prospectively tested, seroreactivity in donors from moderately to highly *Babesia*-endemic counties of New York was considerably lower (0.28%, 38/13,757) [23]. A subset (37/60) of seroreactive donors were prospectively followed for at least 12 months following donation, 20 of 37 (54%) completed the 12-month follow-up visit of whom 15 (75%) were still seroreactive [19]. Nine donors were identified as being PCR-positive during index screening. Of the five donors who participated in the follow-up study, three were PCR positive at six months, and two remained positive at final follow-up at 12 months (378- and 404-day post-index donation). Seropositive donors were questioned whether they had experienced symptoms or signs of *Babesia* infection in the three months prior to donation. Few reported symptoms or signs that were consistent with babesiosis. 

In 2019, the US Federal Drug Administration (FDA) published a guidance for industry that included a non-binding recommendation to perform regional nucleic acid testing (NAT) in 15 high-risk states using licensed assays [53]. At the time of the recommendation, NAT (PCR and transcription mediated amplification) assays had already been licensed for donor screening. NAT has shown high sensitivity and specificity with limits of detection for *Babesia* as low as two to three parasites/mL [54]. The guidance also allows for requalification of those donors who are deferred for a history of babesiosis or positive *Babesia* test result after two years of negative testing. Experimental molecular [17,18] and commercial antibody-based assays [55] have also been developed that could have utility in donor screening. 

In summary, studies of transfusion-transmitted babesiosis highlight persistence of *B. microti* infection with few discernible clinical effects in infected blood donors. Asymptomatic, intermittent and low-level parasitemia can occasionally persist for more than two years. 

## 5. Mechanisms of *Babesia* Persistence

Currently there is a limited understanding of the immune mechanisms that mitigate the severity and duration of *Babesia* infection, however, existing data indicate that innate and adaptive immune mechanisms are able to clear the infection without antimicrobial therapy in the majority of immune competent individuals [10,11,36]. A putative immunological model can be deduced from human and animal studies, and from a review of the large body of work on malaria immunology.

**Figure 3:**
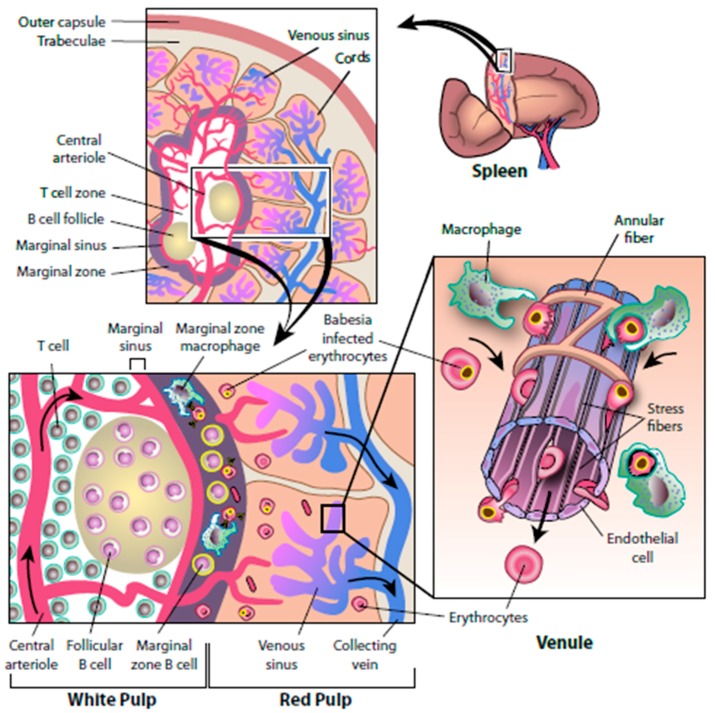
**The splenic response to *Babesia* infection.** The following composite model is based on studies of *Babesia* infection in natural vertebrate hosts and animal models. The spleen is a heavily vascularized organ (top left panel) that consists of red-pulp zones and white pulp zones surrounded by a trabecula and an outer capsule. A circulating erythrocyte travels through the spleen approximately once every 20 min. Erythrocytes enter the spleen by means of the trabecular artery and flow into central arteries and follicular arterioles to reach the marginal sinus of the white pulp. Once in the adjacent marginal zone, *Babesia*-infected erythrocytes are ingested and destroyed by resident dendritic cells and macrophages. Marginal-zone macrophages do not express major histocompatibility complex (MHC) class II molecules but shed pathogen-degradation products that are picked up by marginal-zone B cells. Activated marginal-zone B cells and dendritic cells move to the T-cell zones, where they present antigen to T cells. Activated T cells migrate to the edge of the follicles and engage B cells, causing them to activate and eventually differentiate into antibody-secreting cells. Opsonization of *Babesia*-infected erythrocytes by antibody promotes their clearance by phagocytes. Activated T cells also produce interferon-γ, the prototypic cytokine that helps macrophages kill ingested pathogens. Blood may bypass the white pulp and reach the red pulp directly. In the splenic cords of the red pulp, blood cells slowly flow between reticular fibers and are sensed by resident macrophages. *Babesia*-infected erythrocytes squeeze with difficulty through the apertures of the endothelium lining and are ingested by resident macrophages of the cords. Stress fibers that run longitudinally at the base of the endothelial cell lining and connect to annular fibers can contract and loosen, thereby regulating the flow and size of erythrocytes that reach the venous sinuses. Blood cells that access the venous sinuses flow into venules and eventually reach the collecting vein. (Adapted from [1]).

The initial immunological barrier that *Babesia* encounter after transmission to a human host is the spleen. Individuals who lack a spleen and become infected with *Babesia* generally experience severe infection, which is associated with a high fatality rate [1,5,10,56,57,58]. Many of the earliest reported cases of babesiosis involved asplenic individuals because the severity of those infections prompted a comprehensive diagnostic evaluation. This led to the discovery of *Babesia* parasites during microscopic evaluation of thin blood smears. Dr. Jane Deforges wrote an editorial in 1957 after cases were first described on Nantucket Island (babesiosis was initially known as Nantucket fever), suggesting that those who were asplenic should avoid travel to the Island [59]. Today this admonition would need to include large areas of the Northeast and northern Midwest. Numerous subsequent reports, including both human and animal studies, have demonstrated that the spleen plays a crucial role in the immune response to *Babesia* infection [1,5,57,58,60,61]. After *Babesia* parasites are introduced into the bloodstream following a tick bite, they enter the spleen through the splenic artery, course through the white pulp and empty into the marginal zone blood vessels or the vascular tufts and sinuses in the red pulp (Figure 3). The marginal zone contains macrophages and neutrophils that recognize and ingest *Babesia*-infected red blood cells and circulating *Babesia* parasites. In the red pulp, *Babesia*-infected red blood cells are captured in sieve-like slits in the sinuses as they return to the main circulation and are ingested by macrophages [1,5]. The white pulp of the spleen contains T cells that produce cytokines such as gamma interferon (IFNγ), which enhance macrophage destruction of *Babesia* and activate B cells to secrete *Babesia*-specific antibody. 

As with other aspects of *Babesia* immunity, much can be deduced from studies of malaria immunity. Antibody has been shown to be crucial in reducing malaria parasite load, thereby limiting the severity of disease and helping to prevent subsequent malarial infection. There are thousands of malaria strains so that sterilizing immunity can be achieved against a specific strain but not all strains [7]. Partial immunity from cross-reacting antibody can limit the severity of infection of most malaria strains. People living in malaria-endemic areas generally experience multiple malarial infections, but the disease becomes less severe over time. There is good evidence that antibody is also important in limiting and clearing *Babesia* infection, at least in immunocompromised individuals [12,34]. Patients with B cell lymphoma and/or Rituximab therapy have impaired antibody responses and were found to have a prolonged relapsing clinical course despite antimicrobial therapy [12,62]. Antibody clears infection through neutralization by blocking pathogen entry into red blood cells, enhances opsonization of parasites by macrophages and neutrophils, eradicates pathogens through antibody-dependent cytotoxicity by natural killer cells, and activates complement.

*Babesia* parasites employ a number of evasive measures to avoid an immune attack, which leads to persistent *B. microti* infection, even when immune function is intact [6]. For one, the invasion of red blood cells limits pathogen exposure. Secondly, expression of *Plasmodia* and *Babesia*-induced adhesion molecules on the red blood cell surface have been identified that cause adherence of parasite-infected erythrocytes to vascular endothelium [5,6,63]. As a result, parasites can complete their entire life cycle and invade other erythrocytes without ever circulating through the spleen. The importance of this mechanism is emphasized by the production of antibody against these adherence proteins, which block their action. Great variability exists among the genes that encode these proteins. Antibody directed against one type of adherence protein may be ineffective in blocking a second type of adherence protein. Parasite-induced adherence proteins have been identified for several *Babesia* species, including *Babesia bovis* in cattle, although they have yet to be demonstrated for *B. microti* [6,63,64]. *Babesia* also can develop resistance to antiparasitic drugs, including the two standard combinations of atovaquone and azithromycin and clindamycin and quinine. The molecular origin of *B. microti* resistance to atovaquone and to azithromycin have been identified as genetic mutations in certain *Babesia* strains, which alter the microbial protein targets of the drugs These targets are the cytochrome b protein in the *Babesia* mitochondrial electron transport chain for atovaquone and the apicoplast protein, which inhibits protein translation in this organelle for azithromycin. Atovaquone and azithromycin cannot effectively bind and inhibit the growth of these strains [65,66]. 

Cytokines are another immune component that is thought to play an important role in protecting against *Babesia* infection. They are small signaling molecules of critical importance in normal immune function and other biological processes [67]. Hundreds of different cytokines are secreted by immune and non-immune cells. Cytokines can stimulate or inhibit cell differentiation and proliferation, cell activation, cell migration, and cell survival. Proinflammatory cytokine release helps activate the immune response to eradicate *Babesia* [68,69]. An impaired cytokine response would likely facilitate persistence of *B. microti* infection, although there is no data to support this possibility. Paradoxically, an excessive cytokine response is thought to enhance disease and contribute to complications and death [5,69,70]. A good example is the apparent cytokine-induced pulmonary edema and death following *B. duncani* infection in hamsters. *B.duncani* infected hamsters uniformly have elevated lung concentrations of TNFα and IFNγ. In contrast, *B. microti* infection elicits a mild clinical response in hamsters, and pulmonary TNFα and IFNγ are not upregulated. Further studies of the role of cytokines in the persistence of *Babesia* infections in humans are needed.

## 6. Conclusions

- Persistence of *Babesia* infection in mammalian hosts is an important characteristic of these parasites that maximizes the probability of transmission between vector and host.
- Asymptomatic persistence of *Babesia* infection in blood donors increases the probability of transmission to blood transfusion recipients.
- Persistent asymptomatic *Babesia* infection in blood donors is being addressed through implementation of blood donor screening.
- Symptomatic persistence of *Babesia* infection in immunocompromised hosts increases the probability of disease complications and death.
- The health burden of persistent and recrudescent babesiosis can be minimized by development of novel therapeutic measures, such as new antiparasitic drugs or drug combinations, improved antiparasitic drug duration strategies, or immunoglobulin preparations; and novel preventive approaches, including early detection methods, tick-avoidance, and blood donor screening.
